# Sonochemical degradation of bisphenol A: A synergistic dual-frequency ultrasound approach

**DOI:** 10.1016/j.ultsonch.2025.107488

**Published:** 2025-07-30

**Authors:** Shaun Fletcher, Lukman A. Yusuf, Zeliha Ertekin, Mark D. Symes

**Affiliations:** School of Chemistry, University of Glasgow, Glasgow G12 8QQ, United Kingdom

**Keywords:** bisphenol A, Pollutant degradation, Acoustic cavitation, Wastewater treatment

## Abstract

The persistence of bisphenol A in the environment poses significant ecological hazards. Traditional treatment methods often fall short in removing micropollutants such as bisphenol A from wastewater. The use of ultrasound in water treatment has the potential to induce powerful oxidative degradation of micropollutants while dispensing with the need for chemical intervention. Herein, we show a novel approach for the sonochemical degradation of bisphenol A using dual frequency ultrasound. The synergistic effects of using two distinct ultrasonic frequencies (20 kHz, with the addition of either 37 kHz or 80 kHz) were investigated in the context of bisphenol A removal. The method was shown to substantially increase the rate of degradation compared to single frequency treatment, achieving a 94% removal of bisphenol A under optimised conditions. The high extent of chemical oxygen demand removal and the absence of a requirement for chemical additives demonstrates the promise of this method as a green alternative for water treatment.

## Graphical abstract

**Figure:**
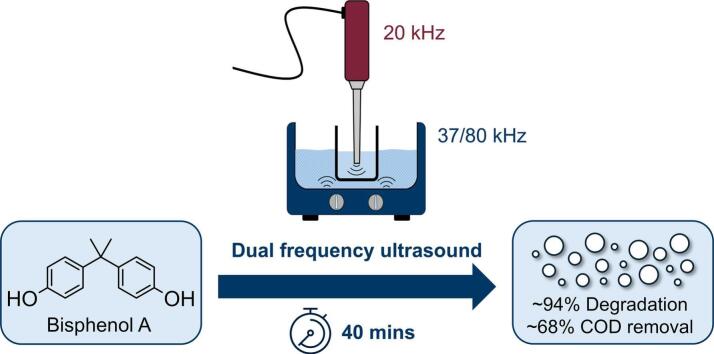

## 1 Introduction

Concerns around the consequences of high levels of micropollutants in the environment (such as pharmaceuticals and industrial chemicals) have motivated intense research into new wastewater treatment methods [[1], [2], [3]]. Bisphenol A (BPA) is one such pollutant which has garnered particular interest in this context, with approximately 10 billion kilograms produced annually for use in plastic manufacturing. As an endocrine disruptor, BPA has been shown to affect the natural balance of hormones in the human body by binding to oestrogen receptors, with potential effects on both adult and foetal development in humans [4]. While its usage in consumer applications (such as food packaging and thermal paper receipts) has been curtailed in recent years, BPA remains an important monomer for the production of polycarbonates and other rigid plastics [5,6]. Its ubiquity means that there are many routes by which BPA can be discharged into the environment, which conventional water treatment plants are not always equipped to deal with [7]. Therefore, to avoid exposure risks on the physiology of both humans and aquatic organisms, robust wastewater treatment processes specifically targeting micropollutants such as BPA are crucial.

Some of the most promising methods for the treatment of recalcitrant pollutants are advanced oxidation processes, whereby contaminants are attacked by reactive oxygen species such as hydroxyl (·OH) and hydroperoxyl (·OOH) radicals. This instigates a cascade of oxidative reactions, resulting in the breakdown of large organic compounds into smaller moieties, which are ostensibly less harmful and have a higher degree of biodegradability [8]. The necessary radicals are generated in situ using oxidants, catalysts, ultraviolet irradiation, ultrasound, or combinations thereof. Of these approaches, the use of ultrasound energy to induce radical formation is particularly appealing, as it is not reliant on the quality of the water, and can be integrated well into existing treatment infrastructure [9].

The propagation of ultrasound through a fluid causes acoustic cavitation: the formation, growth, and rapid implosion of small bubbles formed at nucleation points throughout the medium (dissolved gases, etc.) [10]. The collapse of these bubbles can be modelled as an adiabatic process, whereby gaseous compression leads to intense heating within the bubble, leading to the creation of a highly reactive “hot-spot” [11]. This region, comprising both the bubble and the fluid immediately surrounding it, has enough energy to trigger homolytic splitting of water molecules and dissolved oxygen in aqueous media, forming (among others) the species described in Equations (1), (2), (3), (4).

(1) $$H_{2}O\;\overset{\;\;\;ultrasound\;\;\;}{\to }\;∙H\;+\;∙OH$$

(2) $$O_{2}\;\;\overset{\;\;\;ultrasound\;\;\;}{\to }\;2\;∙O$$

(3) $$H_{2}O\;+\;∙O\;\overset{\;\;\;\;\;\;\;\;\;\;\;}{\to }2\;∙OH$$

(4) $$O_{2}\;+\;∙H\;\overset{\;\;\;\;\;\;\;\;\;\;\;}{\to }\;∙OOH$$

The extent to which these reactive oxygen species form during irradiation with ultrasound is strongly dependent on the nature of the cavitating bubbles formed. Their size and lifetime, and therefore the associated sonochemical properties, can be tuned by manipulating the ultrasonic frequency [12].

The application of sonochemically-driven BPA degradation has been the subject of previous research. However, the majority of these studies focused on enhancing the overall oxidative effect with additives such as hydrogen peroxide [13], persulfate [14] and ferric ions [15,16], as well as hybrid techniques combining ultrasound with photocatalysts and ultraviolet light [17]. While these tandem techniques show considerable improvement in the extent of both BPA degradation and mineralisation (that is, the complete transformation of an organic species into simple inorganic compounds), additives in these processes may also act as secondary pollutants themselves if not properly removed following treatment. Considerably less work has been done to optimise the ultrasonic aspects of the degradation process.

The use of multiple ultrasound sources to induce acoustic cavitation in a fluid has attracted attention in recent research [18]. The interaction of sound waves at differing frequencies in a sonoreactor generates a complex acoustic field, comprising composite sound waves of frequencies including the sum and difference of the incident waves [19]. The cavitation produced by this enriched acoustic field has the potential to yield sonochemical effects greater than the cumulative effect of its constituent parts [20,21]. In this spirit, this study shows that the sonochemical degradation of BPA is improved by the implementation of dual frequency ultrasonic irradiation from discrete sources. This technique is shown herein to improve the effectiveness of acoustic cavitation while dispensing with the need for additional chemical intervention. Coupled with the higher sonochemical efficacy afforded by dual frequencies, this is highly pertinent for applications relevant to recalcitrant pollutant degradation. Hence, in this work, we demonstrate effective degradation of BPA under dual frequency sonication, with particular emphasis on the synergistic effect.

## 2 Experimental

### 2.1 Materials

Bisphenol A (≥99 % purity, Supelco, CAS Number: 80–05-7) was used in all experiments without further purification. For analytical calibration, five solutions with concentrations in the range of 10–30 mg L^−1^ were used. For degradation studies, aliquots of 20 mg L^−1^ (87.6 μM) BPA solution were used as model pollutant samples, which were treated for 10, 20, 30, and 40 min under varying ultrasound conditions. For iodometric dosimetry, potassium iodide (99 %, Alfa Aesar) was used.

### 2.2 Ultrasonic apparatus

Ultrasonic irradiation of BPA solutions was achieved with two distinct devices operating at different frequencies. An Elmasonic P30H ultrasonic bath (120/100 W ultrasonic power effective at 37 kHz/80 kHz, respectively) was used to generate switchable 37 kHz or 80 kHz frequencies. The bath was filled with 600 mL of deionized water, and a glass sonoreactor (built in-house from borosilicate glass, cylindrical in shape, with inner diameter = 50 mm, wall thickness = 3 mm, and height = 68 mm) containing 25 mL of sample was positioned *ca.* 10 mm from the base. In this way, ultrasonic energy was transferred to the sample indirectly via the surrounding water. A Branson 450 Digital Sonifier (400 W) was used to introduce 20 kHz ultrasound irradiation into the solution through a titanium probe (diameter = 6.35 mm, length = 230 mm). The tip of the probe was placed *ca.* 10 mm beneath the surface of the analyte solution to prevent any agitation or aerosoling at the top layer. Two dual frequency regimes, in which the bath operated at i) 37 kHz and ii) 80 kHz (in each case operating alongside the probe at 20 kHz), were used in this work. For the study of BPA degradation, experiments under each ultrasound regime were performed in triplicate. Additional analyses, whereby only one source of ultrasound was used for sonication, were performed to assess the synergistic effect.

It is important to note that the input power of an ultrasonic device does not translate perfectly to the acoustic power dissipated in a sonicated sample. The acoustic power density afforded by the assembly used in this work has been estimated using calorimetry. The temperature of the liquid inside the sonoreactor was recorded for 200 s, with the rate of temperature increase then being used to derive the power density. The data for the relevant groupings of power and frequency are shown in Table 1 [22]. This serves as an approximation for the acoustic power afforded by the apparatus, and does not account for minor variations due to the inhomogeneous acoustic field or heat loss from the free surface. The ultrasonic probe was set at 80% of its maximum power in order to minimize any instabilities in the energy output over extended periods of time [23].

**Table 1:** Acoustic power density delivered by various configurations of the sonication apparatus [22]. The probe was always used at 80% of its maximum rated power and the bath was always used at 100% of its maximum rated power.

**Entry**	**Probe frequency/kHz**	**Bath frequency/kHz**	**Acoustic Power Density/W L^−1^**
1	20	−	146.08 ± 4.83
2	−	37	131.18 ± 2.14
3	−	80	87.03 ± 1.95
4	20	37	272.08 ± 3.92
5	20	80	209.63 ± 6.94

Thermal control of the reactor was necessary to mitigate unwanted heating of the sample during the sonication process. The temperature of a given sample was monitored throughout each experiment with a thermocouple (Pico Technology TC-08) and kept to a maximum of 25 °C with the periodic addition of fresh cold water to the ultrasonic bath. This is analogous to the use of cooling jackets employed in many examples of sonochemical reactors [24,25].

### 2.3 Analytical methods

Measurement of BPA concentration in stock and treated samples was performed with an Agilent 1290 Infinity high performance liquid chromatograph (HPLC) attached to an Agilent 6125B Single Quad mass spectrometer (MS). A Phenomenex 15 cm C18 column was used as the stationary phase. The mobile phase was a 50/50 (v/v) mixture of 0.1 % formic acid in (a) water and (b) acetonitrile (Fisher, HPLC grade), with a flow rate of 0.5 mL min^−1^ and a sample injection volume of 10 μL. An ultraviolet detector monitoring 273 nm was used to identify fractions. The concentration of BPA in each treated sample was determined by the area of the characteristic chromatographic peak (at retention time, rt = 2.3 min) compared to the chromatograms of the stock solutions (see Supplementary Fig. 1). Validation of the transformation products of the sonochemical process was achieved with an Agilent 6546 Q-TOF-MS high resolution accurate mass spectrometer.

To quantify the production of oxidative species by cavitation bubbles, dosimetry with potassium iodide solution was employed [26]. The oxidation of iodide in solution generates molecular iodine (Equation (5). When excess iodide ions are present, molecular iodine forms the triiodide ion (I_3_^−^) (Equation (6).

(5) $${2\text{I}}^{-}\;\overset{\;\;\;\text{oxidation}\;\;\;}{\to }\;{\text{I}}_{2}$$

(6) I_2_ + I^−^ ⇌ I_3_^−^

Triiodide exhibits a strong absorbance peak at 350 nm. In the context of ultrasonic irradiation of KI, the concentration of I_3_^−^ serves as an analogue for the oxidising capability of the system *via* reactive oxygen species generated by the processes in Equations (1), (2), (3), (4). For each of the combinations of ultrasonic bath and horn used in this work, 20 mL of 0.1 M KI solution was subjected to ultrasonic irradiation for 5 min. The concentration of triiodide was determined by measuring the 350 nm absorbance peak of the treated solutions with an Agilent Cary 60 UV–visible spectrophotometer. A calibration of absorbance at 350 nm vs triiodide concentration is provided in Supplementary Fig. 2.

To determine the extent of BPA degradation during ultrasonic treatment, chemical oxygen demand (COD) techniques were used. A photometer (Hanna Instruments, HI-97106) with accompanying reagent test kits (Hanna Instruments, HI-93754D-25) was used to obtain the COD (in mg L^−1^) of treated BPA samples. The COD removal was calculated with Equation (7) by comparing the COD to that of the stock solution.

(7) $$Extent\;of\;\;chemical\;oxygen\;demand\;removal\;=\;1-\left(\frac{COD_{sample}}{COD_{stock}}\right)$$

## 3 Results and discussion

### 3.1 Degradation of bisphenol A and rate analysis

**Fig. 1:**
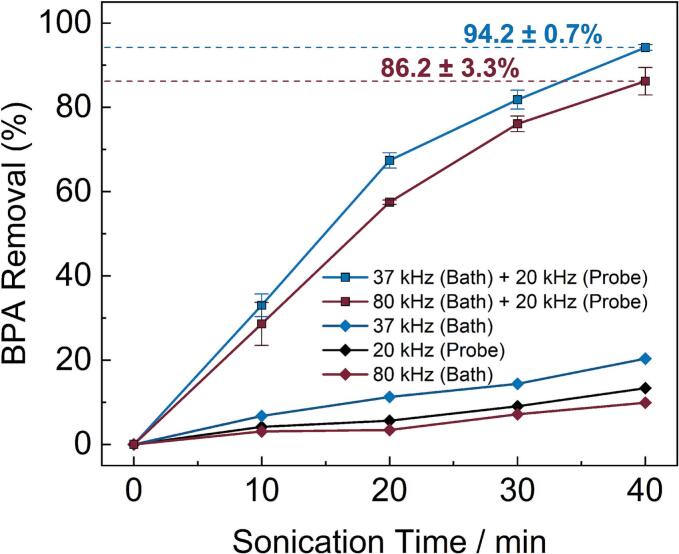
Percentage of bisphenol A removed from solution by dual frequency ultrasound irradiation compared to single frequency treatment as a function of sonication time. The data from the 37/20 kHz (blue squares) and 80/20 kHz (burgundy squares) sonication regimes show a respective 94.2 ± 0.7 % and 86.2 ± 3.3 % removal of bisphenol A after 40 min. The data from single frequency irradiation with 20 kHz (black diamonds), 37 kHz (blue diamonds), and 80 kHz (burgundy diamonds) are plotted on the same scale for comparison. (For interpretation of the references to colour in this figure legend, the reader is referred to the web version of this article.)

The results of experiments investigating the degradation of BPA with dual frequency ultrasound exposure are summarised in Fig. 1. Selected LC-MS chromatograms illustrating the change in BPA concentration with increasing sonication time, from which the degradation data were derived, are shown in Supplementary Figs. 3 and 4. The data show a high conversion of BPA after a 40-minute sonication period for both the 37/20 kHz and 80/20 kHz sonication regimes. A maximum of 94.2 ± 0.7 % removal of BPA was recorded with the 37/20 kHz setup, outperforming the analogous result (86.2 ± 3.3 %) with the 80/20 kHz frequency regime, although it should be noted that the acoustic power obtained with the former is higher than that of the latter.

The sonochemical degradation of BPA follows *pseudo* first-order kinetics [27]. That is, the rate equation can be expressed in the form shown in Equation (8).

(8) $$\mathrm{Rate}\approx -\frac{\mathrm{dC}}{\mathrm{dt}}=kC$$

Here, *C* is the concentration of BPA at time *t*, and *k* is the *pseudo* first-order rate constant. We can therefore determine *k* with the integrated rate equation described in Equation (9).

(9) $$\ln\left(\frac{C_{0}}{C}\right)=kt$$

In this case, *C_0_* is the initial concentration of BPA. Plots of ln(*C_0_*/*C*) vs. sonication time are shown in Supplementary Fig. 5, where the slopes of the curves plotted with linear regression modelling have been used to deduce *k* for each dual frequency ultrasound setup. The data show that the initial rate of BPA degradation is approximately a third greater with 37/20 kHz dual frequencies relative to 80/20 kHz ultrasonics. The superior performance of the lower frequency regime in this instance can likely be attributed to the difference in the acoustic power density offered by each treatment method. The sonochemical effect is governed primarily by the frequency and amplitude of the irradiated ultrasound energy [28]. In general, higher frequency ultrasound requires a greater power amplitude than lower frequencies to achieve a comparable dissipated acoustic power, due to the shorter rarefaction time, smaller resonance size, and overall reduced bubble size. When normalised to the acoustic power density of our equipment (where 37/20 kHz frequencies impart ∼ 30% more power than 80/20 kHz, see Table 1), both have a comparable “per-Watt” rate constant for the degradation of BPA.

### 3.2 Synergistic effect of dual frequency sonication

To ascertain the synergic effects of dual frequency sonication on the degradation of BPA, it is necessary to make the comparison to the situation with single frequency ultrasound irradiation (see Supplementary Fig. 6 for derivations of the *pseudo* first-order rate constants for BPA degradation under single frequency sonication). In this spirit, the synergistic index *S* of sonication with dual frequencies *f*_1_ and *f*_2_ was calculated from the relevant rate constants ($k_{f_{1}f_{2}}$ for dual frequencies, and $k_{f_{1}}$,$k_{f_{2}}$ for single frequencies) for each regime using Equation (10) [17]:

(10) $$S=\frac{k_{f_{1}f_{2}}}{k_{f_{1}}+k_{f_{2}}}$$

The pertinent data is summarised in Table 2, where synergistic indices of 7.1 and 8.2 were calculated in the cases of 37/20 kHz and 80/20 kHz ultrasonic irradiation, respectively.

**Table 2:** Acoustic power density and rate constants for the degradation of bisphenol A with ultrasound, alongside the synergistic index for the dual frequency sonication treatments. See Supplementary Fig. 6 for the derivation of the rate constants for single frequency experiments.

**Frequency/kHz**	**Acoustic Power Density/W L^−1^**	**BPA Degradation Rate Constant/min^−1^**	**Synergistic Index**
20	146.08 ± 4.83	0.0034 ± 0.0002	−
37	131.18 ± 2.14	0.0056 ± 0.0002	−
80	87.03 ± 1.95	0.0025 ± 0.0002	−
37/20	272.08 ± 3.92	0.064 ± 0.004	7.1
80/20	209.63 ± 6.94	0.048 ± 0.002	8.2

The underlying basis of the synergistic effect can be attributed both to the mechanisms of acoustic cavitation in a dual frequency ultrasonic field, and the improved mass transport effects offered by auxiliary ultrasonic irradiation [29]. The motion of cavitating bubbles within an applied sound field are governed by Bjerknes forces. Bubbles can experience attractive and repulsive forces from the pressure nodes of both the incident sound waves (primary Bjerknes forces) and the acoustic radiation emitted by oscillating bubbles (secondary Bjerknes forces) [30]. These dynamisms allow for two bubbles to come together and combine to form a larger bubble, a phenomenon known as coalescence. Numerical modelling of inter-bubble interactions has shown that under certain acoustic conditions, secondary Bjerknes forces exhibit a repulsive effect between bubbles with differing sizes [31]. During irradiation of a fluid with two disparate ultrasound sources, bubbles are formed in high spatial density across a range of sizes determined by the applied frequencies [32]. The distinct driving frequencies (20 kHz and 37/80 kHz) are expected to produce bubbles with different resonance characteristics and oscillation modes. The mismatch in their natural frequencies and phase can lead to asynchronous oscillations, and hence, repulsive secondary Bjerknes forces which inhibit coalescence [33]. The effect of dual frequency also promotes the creation of a greater number of active bubbles, as additional nucleation sites are readily formed within the irradiated sample [22].

In this work, all ultrasonic frequencies used for the treatment of BPA lie in the lower echelons of ultrasonic classification (given that 20 kHz is generally regarded as the threshold for the definition of ultrasound). Bubbles generated at these frequencies undergo violent collapse, generating the intense conditions required for the production of reactive oxygen species. For the amplitude of irradiation by the ultrasonic probe used in this work, we have previously characterised the properties of the resulting acoustic bubbles [23]. It was observed that bubbles generated at the probe tip oscillated in a stable-inertial manner. These bubbles exhibited periodic collapses at integer multiples of the period of the excitation frequency *T_0_* (the exact value is dependent on the excitation amplitude, which in the case of 80% amplitude as used in this work is 5*T_0_*). This suggests that the driving pressure amplitude was below the Blake threshold, leading to stable-inertial cavitation [34]. This mode of cavitation is dominated by both oscillatory dynamics and inertial collapse. Despite the presence of multiple bubbles in the acoustic field, the combination of sub-Blake threshold conditions and frequency-induced phase mismatch makes bubble coalescence unlikely under the specific experimental conditions in this work. This in turn facilitates greater bubble radii growth under dual-frequency ultrasound, as reported in the literature with mathematical modelling [35]. The cavitation dynamics modelling also highlighted increased pressure and water vapour presence within bubbles as a synergistic consequence of dual frequency excitation. These factors all contribute to the quality of the stable-inertial cavitation bubbles under these acoustic conditions. Bubbles formed under the dual frequency regime are more likely to reach the point of collapse [36]. This renders the bulk fluid an ideal medium for the treatment of micropollutants, due to the improved availability of reactive species, through which advanced oxidative degradation can occur. In addition to the enhanced cavitation, it has been proposed that degradation kinetics can be bolstered by dual frequency irradiation due to improved mass transport effects offered by multiple sources of acoustic streaming [37].

### 3.3 Iodide dosimetry and effect of reactive oxygen species

**Fig. 2:**
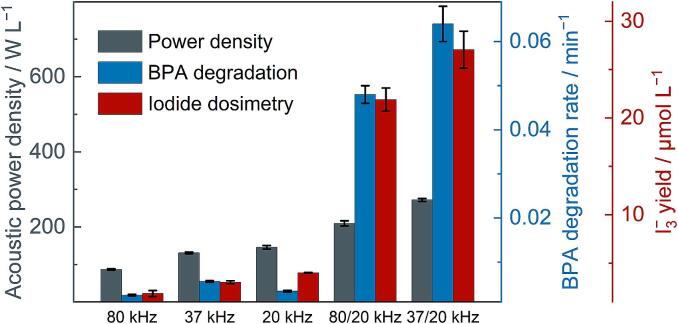
Comparison of the generation of reactive oxygen species with degradation performance. The acoustic power density (grey), BPA degradation rate (blue), and yield of triiodide after 5 min from dosimetry experiments (red) are shown for each experimental setup. A maximum yield of 27.1 ± 1.9 µmol L^−1^ of triiodide was obtained using 37/20 kHz dual frequencies. The use of 80/20 kHz dual frequencies yielded 21.9 ± 1.2 µmol L^−1^ of triiodide. (For interpretation of the references to colour in this figure legend, the reader is referred to the web version of this article.)

A strong synergistic effect when the ultrasonic probe and bath were used simultaneously for the degradation of BPA has been identified. To assess the association of this result with the production of reactive oxygen species (·OH, ·OOH), iodide dosimetry was carried out with the ultrasonic reactor. 25 mL of 0.1 M KI solution was subjected to sonication for 5 min (following the power and frequency used for BPA degradation experiments). Following this, the concentration of triiodide formed by the oxidation of iodide ions was recorded spectrophotometrically (the corresponding UV–visible spectra are displayed in Supplementary Fig. 7). A comparison of the sonochemical yield of triiodide from each experimental regime, together with the acoustic power density and the degradation rate of BPA is shown in Fig. 2.

**Fig. 3:**
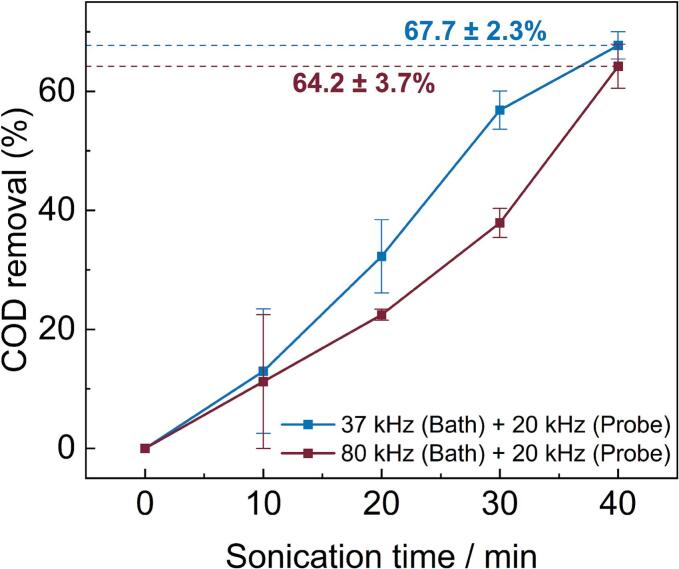
Percentage COD removal from bisphenol A solution by dual frequency ultrasound as a function of sonication time. The data from both 37/20 kHz (blue) and 80/20 kHz (burgundy) sonication regimes are shown, with 67.7 ± 2.3 % and 64.2 ± 3.7 % of COD removed, respectively. (For interpretation of the references to colour in this figure legend, the reader is referred to the web version of this article.)

**Fig. 4:**
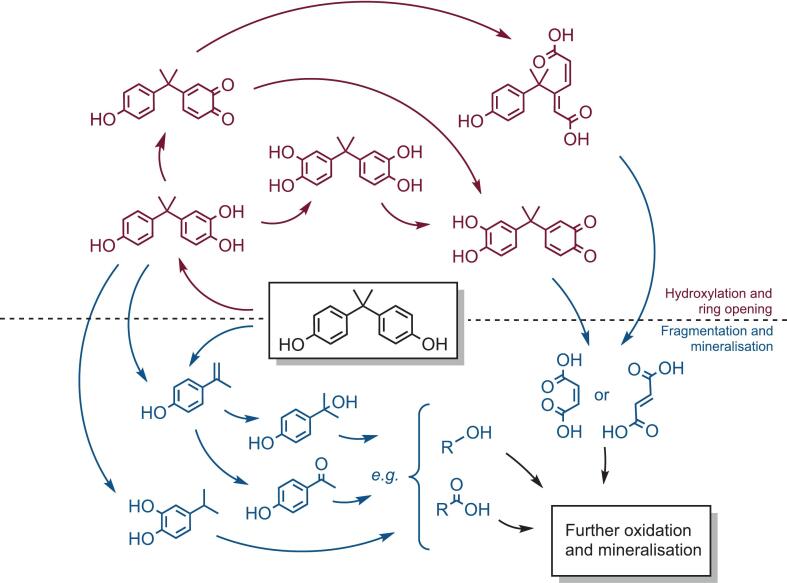
A proposed sonochemical degradation pathway for bisphenol A under dual frequency irradiation, derived from experimental mass spectrometry peaks and reported mechanisms in the literature. The intermediate species are grouped generally into hydroxylation processes (radical mechanisms, shown in burgundy) and fragmentations (thermally-driven mechanisms, shown in blue). (For interpretation of the references to colour in this figure legend, the reader is referred to the web version of this article.)

The data shows a correlation between the effectiveness of a given ultrasonic regime in degrading BPA and the amount of iodide oxidised during dosimetry. This suggests that the rate of degradation of BPA is strongly correlated to the concentration of reactive oxygen species. The figure also highlights the significance of the synergistic effect of dual frequencies, with a pronounced increase in both reactive oxygen species formation and BPA degradation with a modest increase in acoustic power.

### 3.4 Chemical oxygen demand removal

The above results show that dual frequency ultrasound is an effective method for degrading BPA, but it is useful to quantify the amount of organic carbon completely oxidised in a given sample during the experiment [38]. Chemical oxygen demand (COD) is an important metric in the wastewater treatment industry, which is used to quantify the extent to which organic matter is converted to small, highly oxidised fragments. In this work, COD analysis was used to understand the extent of organic carbon oxidation in samples across the period of ultrasound exposure. For each of the dual frequency ultrasound regimes used in this work, the COD of each treated BPA sample was obtained, and compared to the COD of the initial BPA solution. Fig. 3 shows the COD removal of each treatment method. As with the extent of degradation, the system incorporating 37/20 kHz ultrasound exceeds the performance of that afforded by 80/20 kHz frequencies in terms of COD removal. Total COD reductions of 67.7 ± 2.3 % and 64.2 ± 3.7 % were observed from the samples over 40 min by 37/20 kHz and 80/20 kHz frequencies, respectively. Initially, 37/20 kHz dual frequencies show a higher rate of COD removal compared to 80/20 kHz, with both regimes showing comparable results after extended reaction times.

The high level of COD removal from BPA solutions in a dual frequency sonoreactor is likely due to a combination of increased radical prevalence, and a greater extent of thermal degradation. The high local temperatures reached during the rapid collapse of a cavitating bubble can cause thermolysis of pollutant molecules within the bubble or at the bubble–liquid interface [39]. This is strongly dependent on the nature of the pollutant, particularly its solubility and equilibrium vapour pressure. Cavitation bubbles generated by low-frequency ultrasound are generally larger due to the longer rarefaction phase of the acoustic cycle [40]. As a result, these larger bubbles collapse more violently, producing “hot-spots” with higher localized temperatures. This intense collapse can also transfer energy and reactive species further into the bulk fluid. Due to the reduced extent of bubble coalescence afforded by dual frequencies, bubble collapse is much more efficient. It is therefore likely that thermally driven steps in the degradation pathway of pollutant molecules are more readily accessible.

### 3.5 Bisphenol A degradation pathway

The mechanism of the degradation of BPA by advanced oxidation processes has been theorised across the literature; the indiscriminate nature of free radical processes leads to a cascade of reactions with nominal regioselectivity, leading to a wide variety of intermediates and products. The first step is generally accepted as the hydroxylation of the aromatic ring at the position *ortho* to the hydroxy group of BPA [41]. This occurs in parallel to fragmentation across the propylidene group linking the phenolic rings. At this point, many different routes and intermediates have been proposed, involving varying degrees of hydroxylation, oxidation, ring opening, and fragmentation. The upshot is the breakdown of BPA into simple alcohols or carboxylic acids which are subsequently mineralised to inorganics. In this work, mass spectrometry of sonochemically treated BPA solutions was used to rationalise the mechanism of degradation *via* the identification of key intermediates in the process (see Supplementary Fig. 8). The analysis confirmed the presence of monohydroxylated BPA following sonication, alongside further degradation products previously reported. The main species that were observed are reported in Table 3, together with citations to other works in which these species have been identified as intermediates in advanced oxidative degradation of BPA.

**Table 3:** The degradation products of bisphenol A from advanced oxidation processes reported in the literature, all of which were also detected by mass spectrometry analysis in this work.

Structure	Name	M_r_	Found in/proposed by
	Bisphenol A	227.29	−
	Monohydroxylated bisphenol A	244.29	[27,38,41,46,47]
	Dihydroxylated bisphenol A	260.29	[27,38,46,47]
	o-quinone of hydroxylated bisphenol A	242.27	[38,46,47]
	o-quinone of dihydroxylated bisphenol A	258.27	[38,46,47]
	“Ring opened” bisphenol A	276.29	[38,46]
	4-isopropenylphenol	134.18	[27,38,41,46]
	4-hydroxyacetophenone	136.15	[27,38,41,46]
	4-(2 isopropanol) phenol/2-hydroxyl, 4-(isopropyl) phenol	152.19	[38,46]
	Maleic acid/fumaric acid	116.07	[38]

Notably absent from the compiled data were phenol, hydroquinone, or benzoquinone; products which have been reported to form alongside 4-*iso*propenylphenol after hydroxyl radical induced cleavage of BPA [15]. It is likely that these compounds, in addition to other intermediary species with low molecular weights, are more susceptible to complete oxidation *via* thermal degradation. The low volatility of BPA and the associated degradation products suggest that exposure to the sonochemical “hot-spot” occurs at the bubble–liquid interface [39,42]. Due to the low frequency of the ultrasound used in this study (especially from the 20 kHz probe), the “hot-spot” afforded by cavitating bubbles is expected to be intense. This is conducive to an environment throughout the liquid by which efficient degradation of small by-products occurs. This, in turn, rationalizes the considerable extent of oxidation of BPA with the treatment method. The likely pathway for BPA degradation and mineralisation by dual frequency ultrasound is therefore presented in Fig. 4, with the routes between the key species grouped into radical processes (whereby hydroxylation of BPA occurs, yielding intermediary products with higher molecular weights) and fragmentations (species which occur as a result of a combination of thermal and radical mechanisms).

The toxicity of any byproducts formed during the degradation process is an important consideration. The non-selective nature of advanced oxidation processes could lead to the formation of compounds with an equal or greater potential to cause harm [43]. Previous studies have shown that the resultant effluent from electro-Fenton oxidation of BPA is less toxic than the original sample [44]. Similarly, a Fenton-UV process has been shown to eliminate the inhibitory effect of BPA on a sample of *aliivibrio fischeri* [45]. The toxicity was found to decrease with increasing BPA conversion. Many of the byproducts of the Fenton/Fenton-UV process are shared with those yielded by sonochemical degradation. In particular, the lower molecular weight compounds (carboxylic acids, etc.) are thought to be more easily degradable in the environment [41]. Consequently, there is no reason to think that the use of dual frequency ultrasound for BPA degradation should necessarily present any greater ecological risk when compared to other advanced oxidation processes.

### 3.6 Literature comparisons

The results obtained in this work from the dual frequency ultrasound treatment of BPA in water are presented in Table 4 for evaluation against similar studies. In the context of the rate of BPA removal (without consideration as to the nature of the products obtained), dual frequency ultrasonic treatment outperforms similar methods with a single source of irradiation, and is comparable to the results obtained with significant chemical intervention (in particular with Fenton-like processes [16], peroxides [13], and salts [49]). The key mechanism by which pollutants such as BPA are degraded in an advanced oxidation process is by reactive oxygen species generated *in situ*. The works cited in Table 4 utilise the addition of gases, photocatalysts, or electrocatalysts to complement the production of hydroxyl radicals during the sonication process. This work shows that such a boost in the production of reactive oxygen species is achievable through control of ultrasonic parameters. By creating an environment inside the sonoreactor where cavitation bubbles are less likely to coalesce, a greater yield of active radical species can be obtained. Hence, an enhanced degradative effect is observed during BPA treatment. In terms of organic carbon removal, the extent of COD reduction by dual frequency ultrasound treatment is again on a level with that found with chemically-induced intense oxidative processes.

**Table 4:** The degradation of BPA via sonochemical and sono-assisted methods as reported in the literature.

**Ultrasound frequency/** **Reactor volume/** **Acoustic power**	**Additives**	**Starting BPA concentration/mg L^−1^**	**BPA removed (%)**	**Rate constant/min^−1^**	**Organic carbon removed (%)**	**Ref**
500 kHz, 50 mL, 120 W (input)	O_2_ saturated	50	∼95 % after 150 mins	0.0438	(not reported)	[49]
500 kHz, 50 mL, 120 W (input)	Ar saturated	50	∼95 % after 210 mins	0.021	(not reported)	[49]
300 kHz, 300 mL, 80 W (input)	FeSO_4_ (100 μmol L^−1^), UV (254 nm, 25 W)	26.94	∼95 % after 30 mins	(not reported)	∼95 % after 180 mins (COD)	[16]
300 kHz, 300 mL, 80 W (input)	O_2_ gas	26.94	∼100 % after 90 min	0.0339	∼50 % after 540 mins (COD)	[41]
20 kHz, 50 mL, 80 W cm^−2^ (measured)	−	22.83	∼55 % after 60 min	0.007	(not reported)	[27]
20 kHz, 50 mL, 60 W cm^−2^ (measured)	CCl_4_ (25 μg L^−1^)	22.83	∼70 % after 120 mins	(not reported)	(not reported)	[22]
20 kHz, 50 mL, 60 W cm^−2^ (measured)	O_3_ (10 mL min^−1^)	22.83	∼100 % after 60 mins	0.0482	(not reported)	[22]
800 kHz, 200 mL, 100 W (input)	H_2_O_2_ (0.1 mmol L^−1^), pH 7.0 (buffered)	1	∼100 % after 60 mins	0.0908	(not reported)	[13]
580 kHz, 1000 mL, 200 W (input)	Seawater simulant, pH 7.5	0.228	∼93–97 % after 30 min	0.114	(not reported)	[48]
580 kHz, 100 mL, 50 W (measured)	−	25	∼38 % after 60 mins	(not reported)	(not reported)	[46]
580 kHz, 100 mL, 50 W (measured)	CCl_4_ (∼400 mg L^−1^)	25	∼70 % after 15 mins	(not reported)	∼35 % after 15 mins (COD)	[32]
580 kHz, 100 mL, 50 W (measured)	FeSO_4_ (0.1 mg L^−1^)	25	∼72 % after 60 mins	(not reported)	∼24 % after 15 mins (COD)	[32]
580 kHz, 100 mL, 50 W (measured)	Ethyl anthraquinone (0.075 mg L^−1^)	25	∼72 % after 60 mins	(not reported)	∼32 % after 15 mins (COD)	[32]
37/20 kHz, 25 mL 272.08 W L^−1^ (measured)	−	20	∼94 % after 40 mins	0.048	∼68 % after 40 mins (COD)	This work
80/20 kHz, 25 mL 209.63 W L^−1^ (measured)	−	20	∼86 % after 40 mins	0.064	∼64 % after 40 mins (COD)	This work

## 4 Conclusions

This work has demonstrated the synergistic effect of dual frequency ultrasound on the degradation of BPA and has highlighted the use of dual frequency ultrasound as a sustainable and effective method for the removal of refractory micropollutants. The high removal rate of BPA with 37/20 kHz and 80/20 kHz ultrasonic irradiation (*ca.* 94% removal within 40 min in the case of the former) could allow for the effective treatment of polluted water without the need for additional chemical species, and with relatively short process times. The notable level of COD removal that dual frequency treatment offers (up to 68% within 40 min) is also pertinent to wastewater treatment, where removal of organic pollutants is vital. The sonochemical mechanisms by which these statistics are possible have been rationalised on the basis of the improved quality of acoustic cavitation during treatment in terms of bubble size and distribution. The reduced likelihood of bubble coalescence, combined with an increase in the number of active bubbles, is hypothesised to enhance the number and intensity of sonochemical "hot-spots." This in turn is postulated to allow for more effective thermal degradation of BPA, in addition to facilitating the oxidative pathway. Given the importance of preventing the adverse effects of micropollutant contamination, this study shows promise for a sustainable water treatment technology. Further research on optimizing parameters and exploring broader applications in micropollutant degradation are currently underway in our laboratory.

## Data availability

The data underpinning this study have been deposited in the University of Glasgow’s Enlighten database under accession code http://dx.doi.org/10.5525/gla.researchdata.2027.

## CRediT authorship contribution statement

**Shaun Fletcher:** Writing – original draft, Methodology, Investigation, Data curation. **Lukman A. Yusuf:** Writing – review & editing, Methodology, Investigation. **Zeliha Ertekin:** Writing – review & editing, Methodology, Investigation. **Mark D. Symes:** Conceptualization, Supervision, Writing – review & editing.

## Declaration of competing interest

The authors declare that they have no known competing financial interests or personal relationships that could have appeared to influence the work reported in this paper.
