# Supplementary material for: Sonochemical degradation of bisphenol A: A synergistic dual-frequency ultrasound approach
Source: Ultrason Sonochem. 2025 Jul 30;120:107488. doi: 10.1016/j.ultsonch.2025.107488 (PMC12341535; doi:10.1016/j.ultsonch.2025.107488)
Supplement: Supplementary Data 1 [file mmc1.docx]

**Sonochemical degradation of bisphenol A: a synergistic dual-frequency ultrasound approach**

*Shaun Fletcher,^a^ Lukman A. Yusuf,^a^, Zeliha Ertekin,^a^ and Mark D. Symes*^a^*

*^a^WestCHEM, School of Chemistry, University of Glasgow, Glasgow, G12 8QQ, United Kingdom*

**Email:* [*mark.symes@glasgow.ac.uk*](mailto:mark.symes@glasgow.ac.uk)

**Supplementary Information**

*
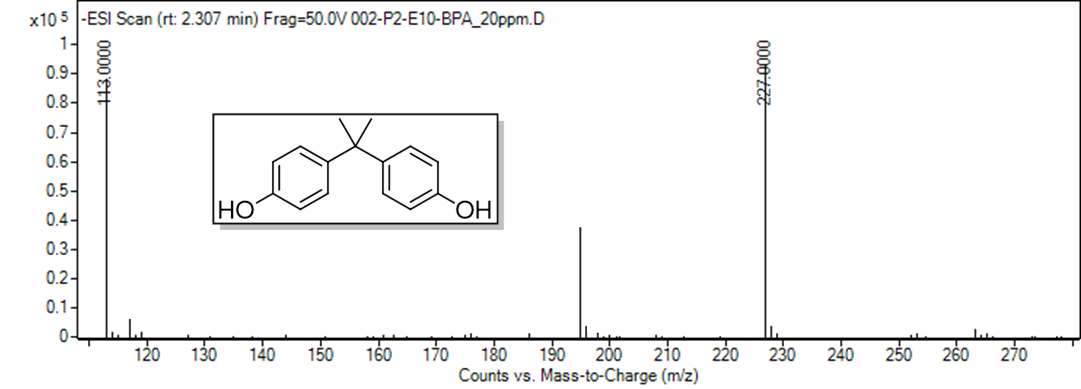
*
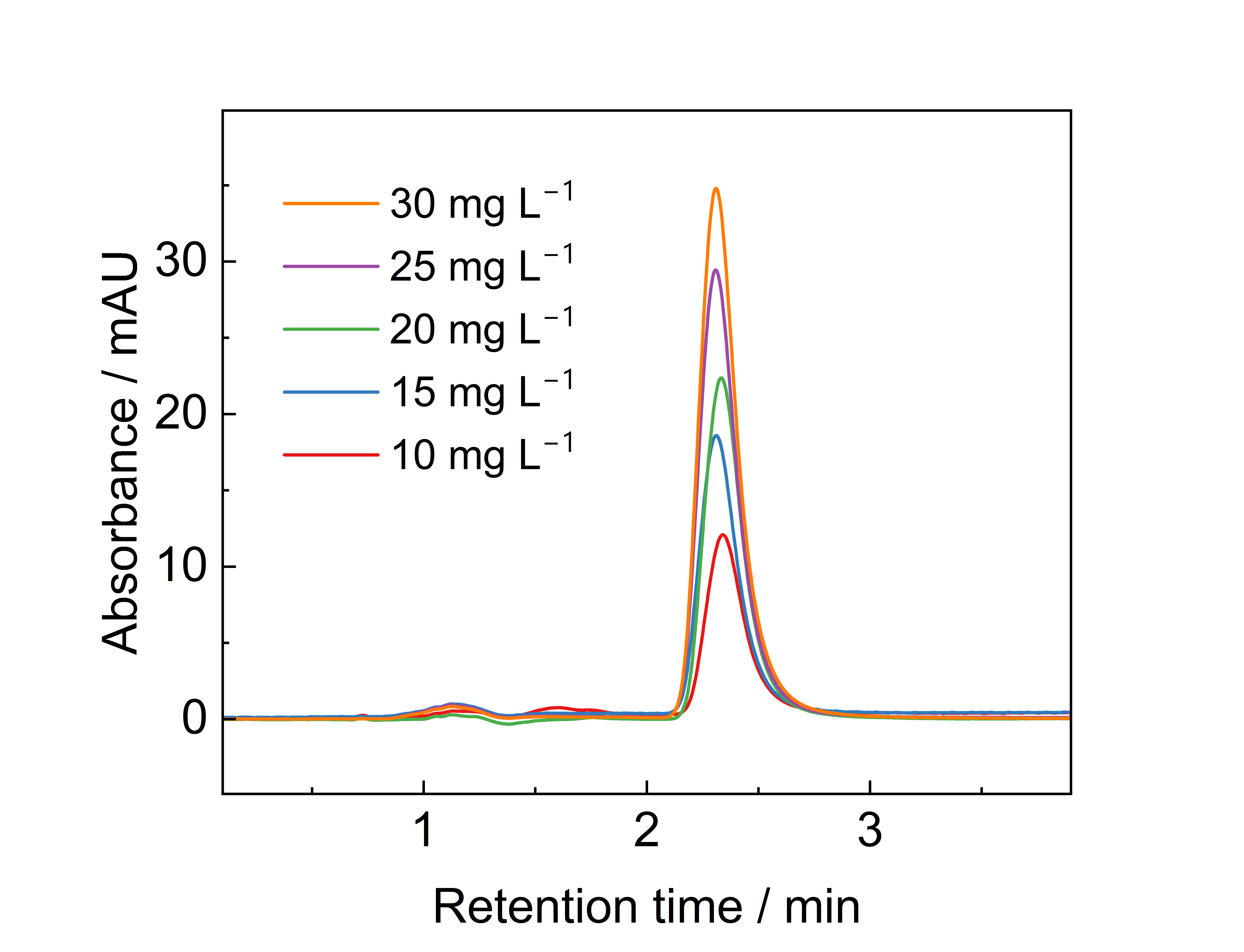

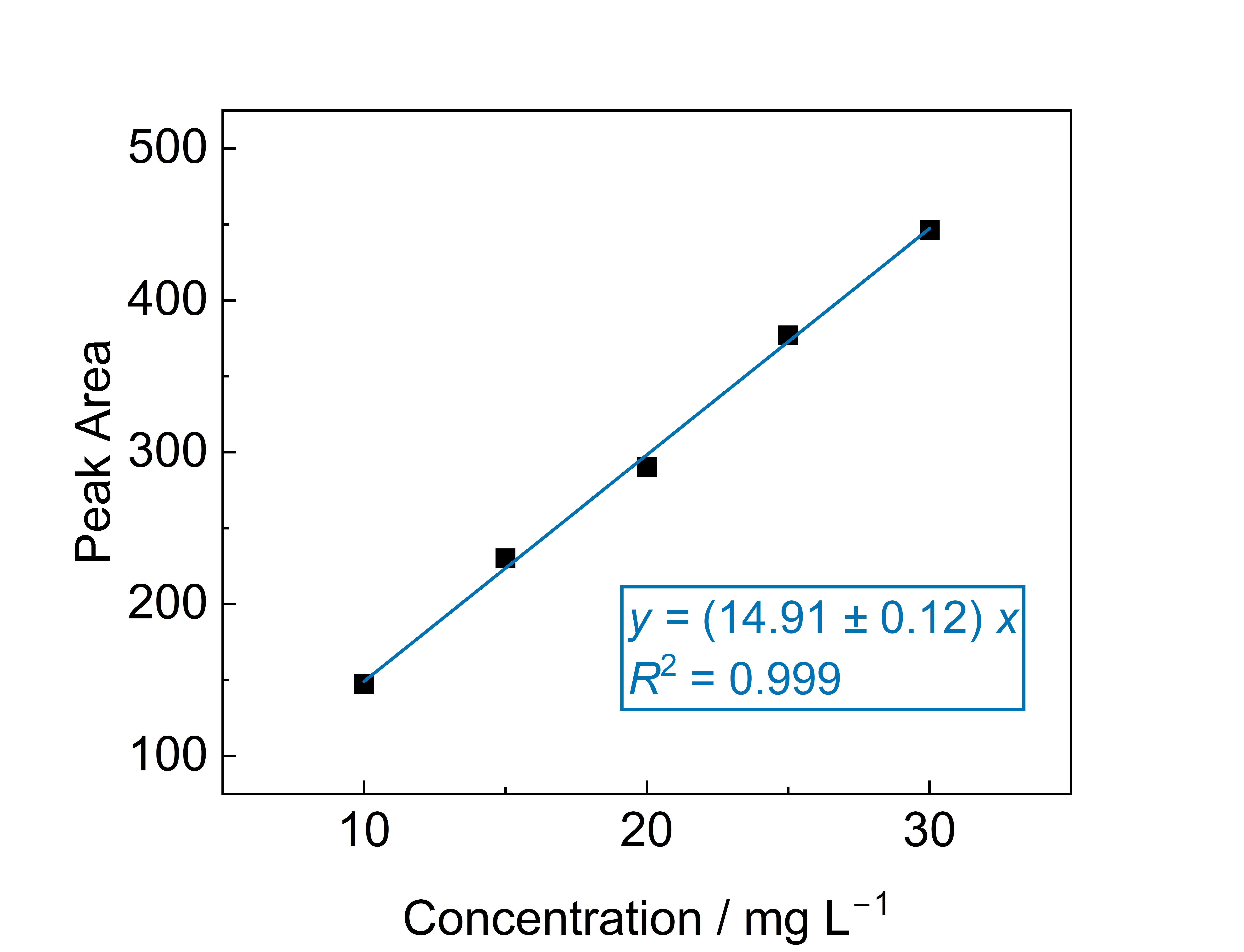


**(a)**

**(b)**

**(c)**

**Supplementary Figure 1:** *Calibration of BPA analytical methods.*

*LC-MS calibration for quantitative BPA analysis: HPLC chromatograms of 10 mg L^−1^ (red), 15 mg L^−1^ (blue), 20 mg L^−1^ (green), 25 mg L^−1^ (purple) and 30 mg L^−1^ (orange) solutions of BPA with absorbance at λ = 273 nm are plotted vs. retention time (a). The area of the characteristic peak (rt = 2.3 min) is plotted vs. concentration of BPA, with linear regression used to define the relationship (b). The corresponding mass spectrum for the characteristic peak of the chromatogram shows signals for both the singly deprotonated ([M-H]^−^, m/z = 227) and doubly deprotonated ([M-2H]^2−^, m/z = 113) molecular ion of BPA (c).*

*
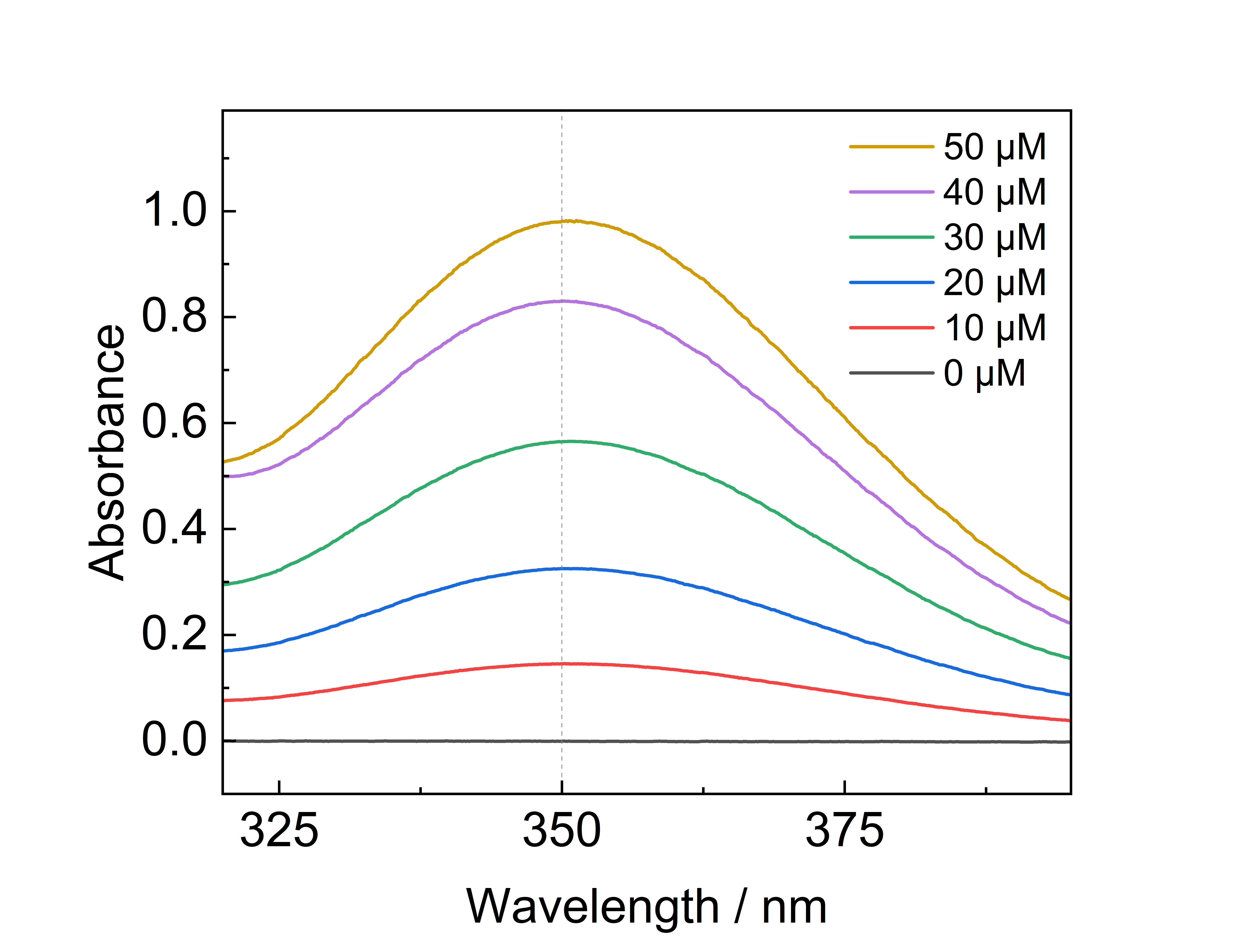
*

**(a)**

*
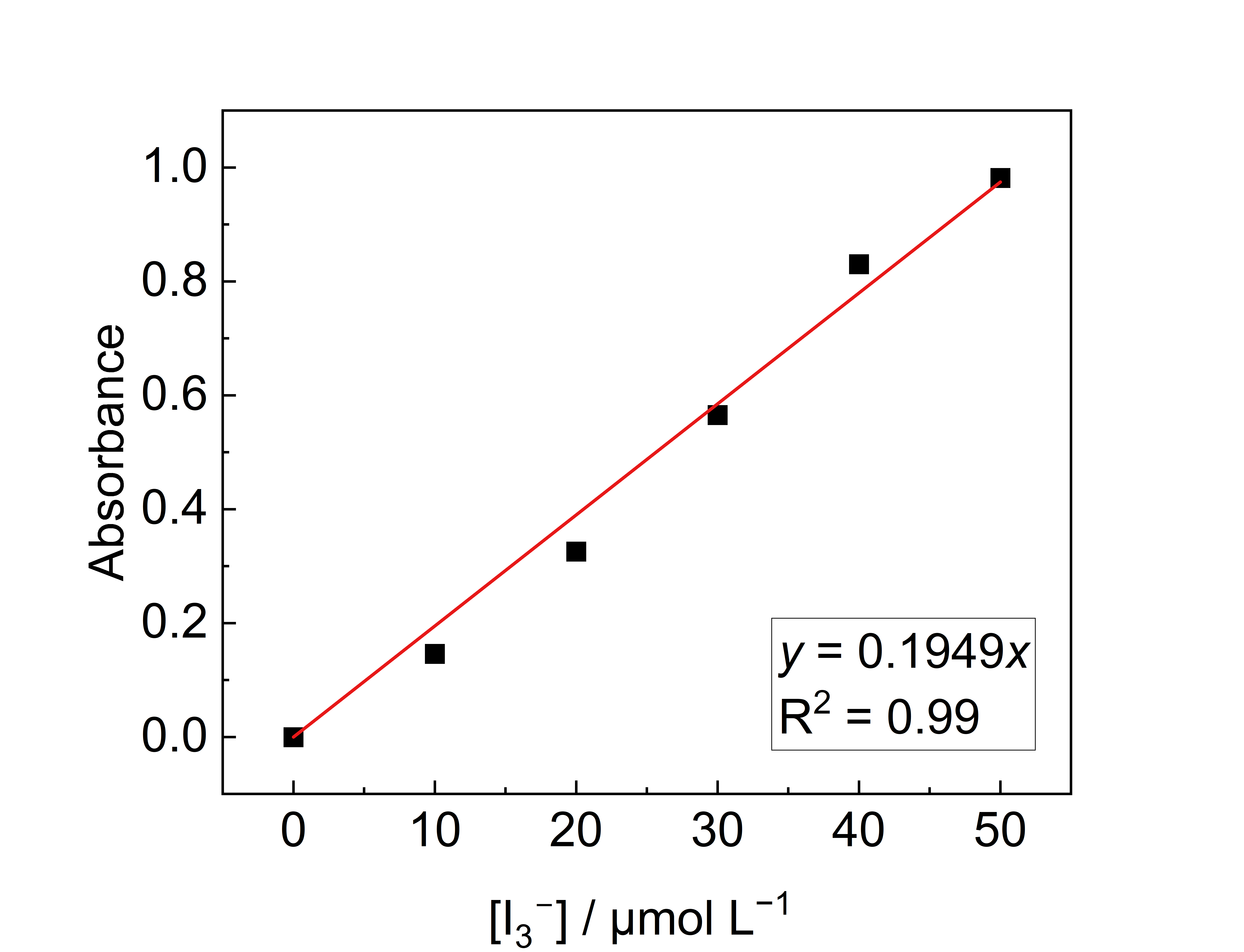
*

**(b)**

**Supplementary Figure 2:** *Calibration of* *iodide dosimetry***.**

*UV-visible spectroscopy calibration for triiodide: UV-visible spectra of stock solutions of triiodide (a). The absorbance at λ = 350 nm is plotted vs. concentration of triiodide, with linear regression used to define the relationship (b).*


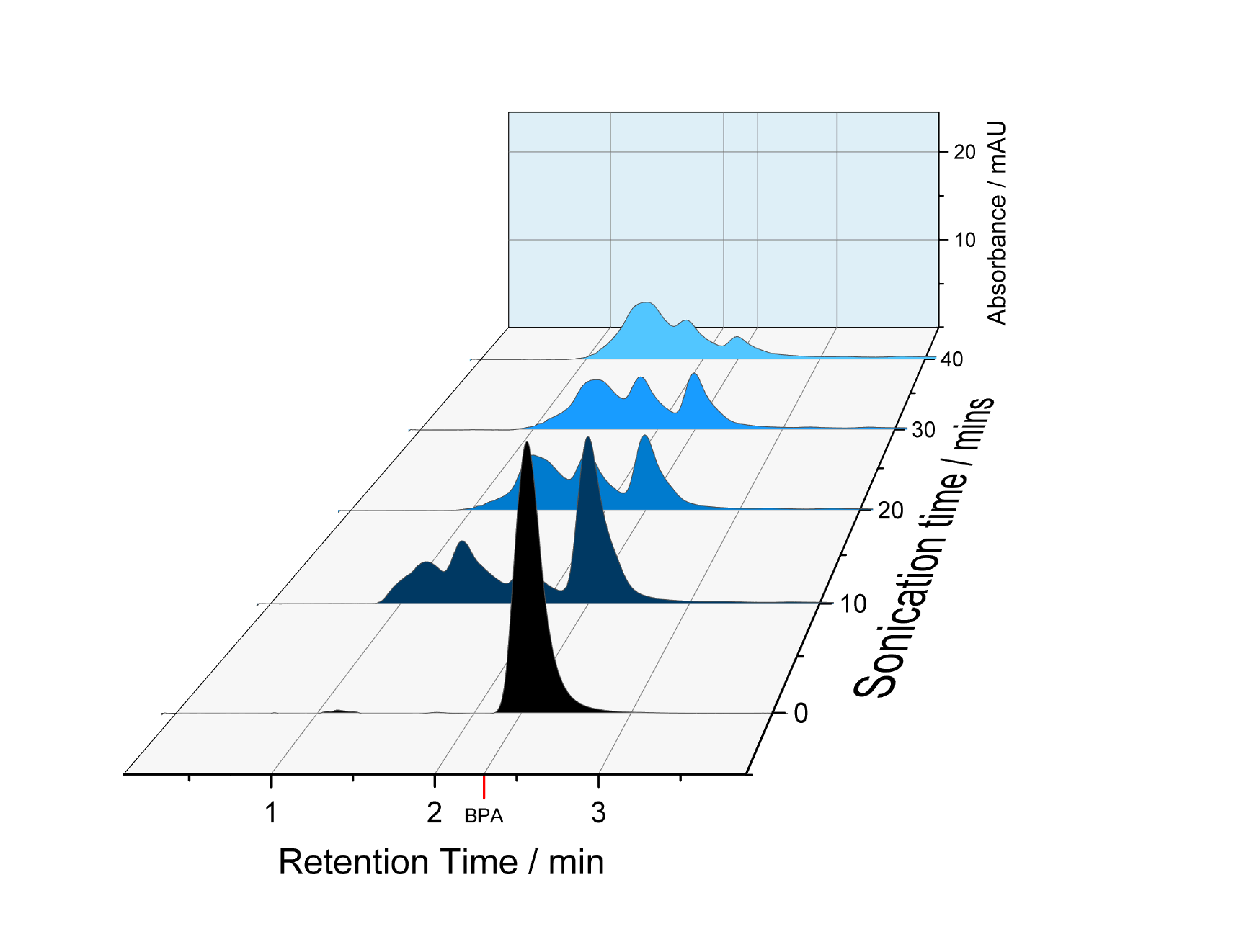


**Supplementary Figure 3:** *Chromatograms of degraded bisphenol A (37/20 kHz).*

*Representative* *HPLC chromatograms of 20 mg L^−1^ BPA solutions following treatment with 37/20 kHz dual frequency ultrasound. Absorbance at λ = 273 nm is plotted vs. retention time in the case of five samples treated for 0 – 40 mins, with chromatograms presented in series with increasing sonication time. The grid line at rt = 2.3 min is provided as a reference for the characteristic chromatographic peak of BPA.*


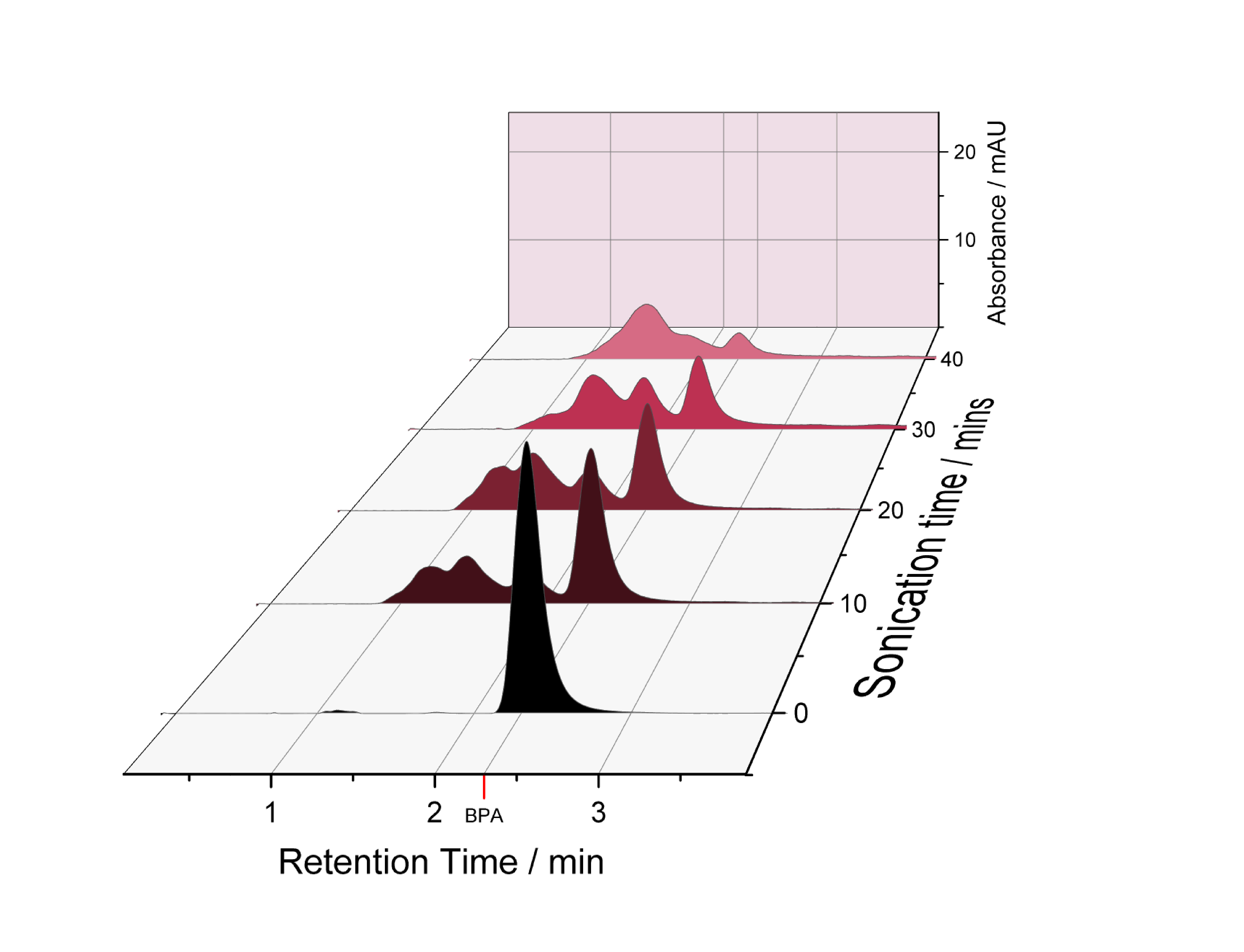


**Supplementary Figure 4:** *Chromatograms of degraded bisphenol A (80/20 kHz).*

*Representative* *HPLC chromatograms of 20 mg L^−1^ BPA solutions following treatment with 80/20 kHz dual frequency ultrasound. Absorbance at λ = 273 nm is plotted vs. retention time in the case of five samples treated for 0 – 40 mins, with chromatograms presented in series with increasing sonication time. The grid line at rt = 2.3 min is provided as a reference for the characteristic chromatographic peak of BPA.*


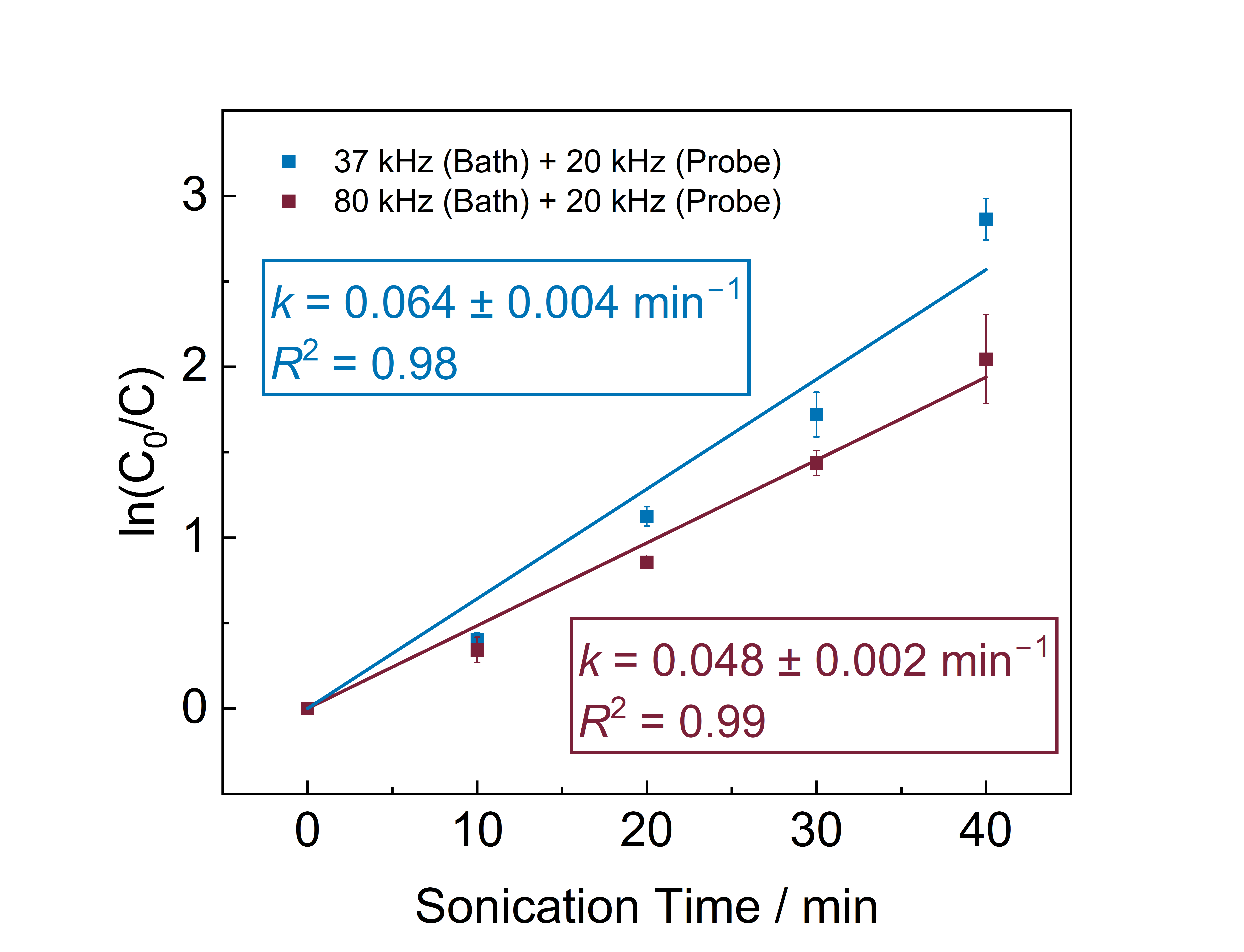


**Supplementary Figure 5:** Rate analysis of bisphenol A removal.

Pseudo first-order rate analysis for the removal of bisphenol A by dual frequency ultrasonic treatment. Rate constants have been calculated as the slope parameter of linear regression modelling of ln (C_0_/C) versus time in each case, determined to be 0.064 ± 0.004 min^−1^ and 0.048 ± 0.002 min^−1^ for treatment with 37/20 kHz (blue) and 80/20 kHz (burgundy) frequencies, respectively.

*
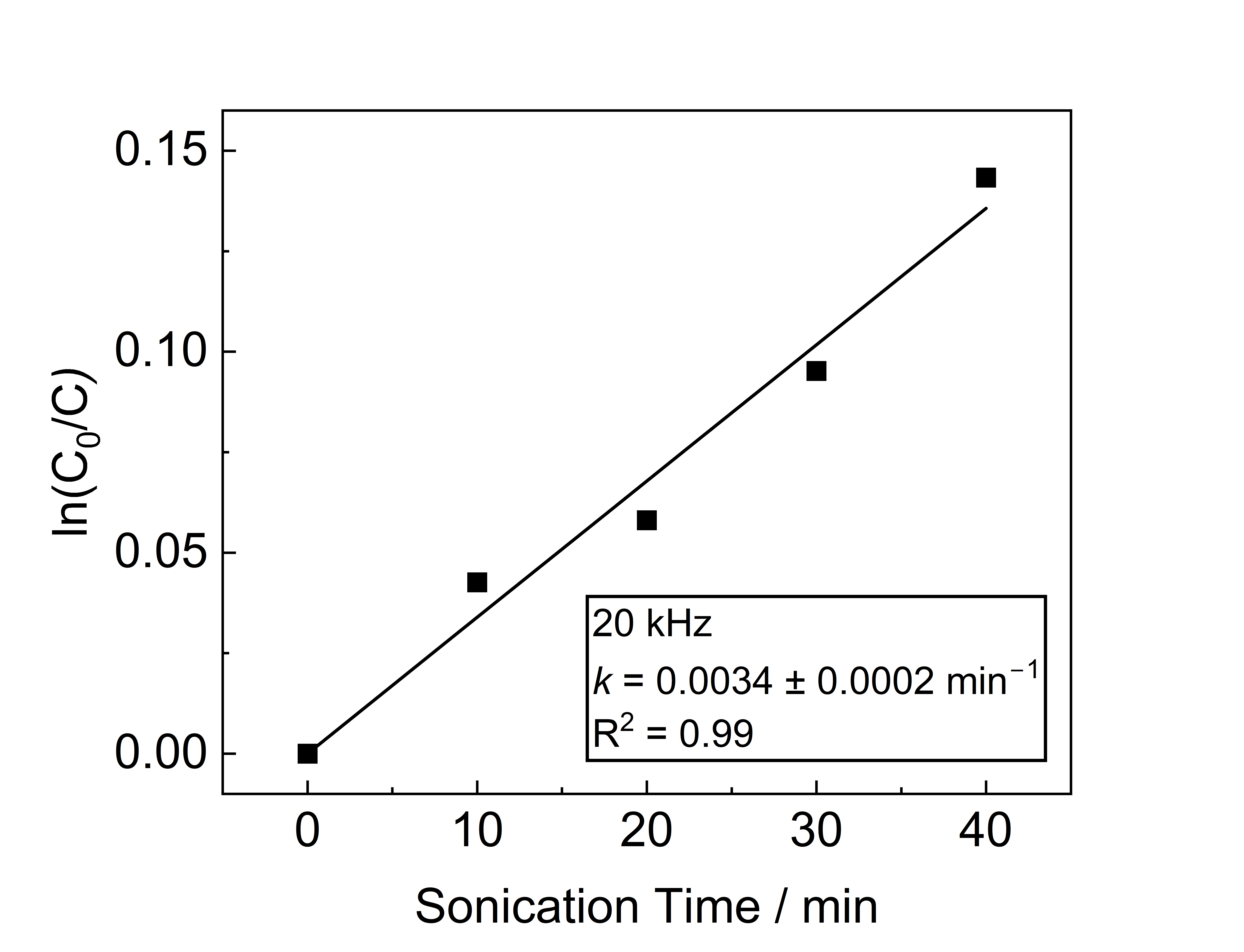
*

**(a)**

**(b)**

**(c)**

*
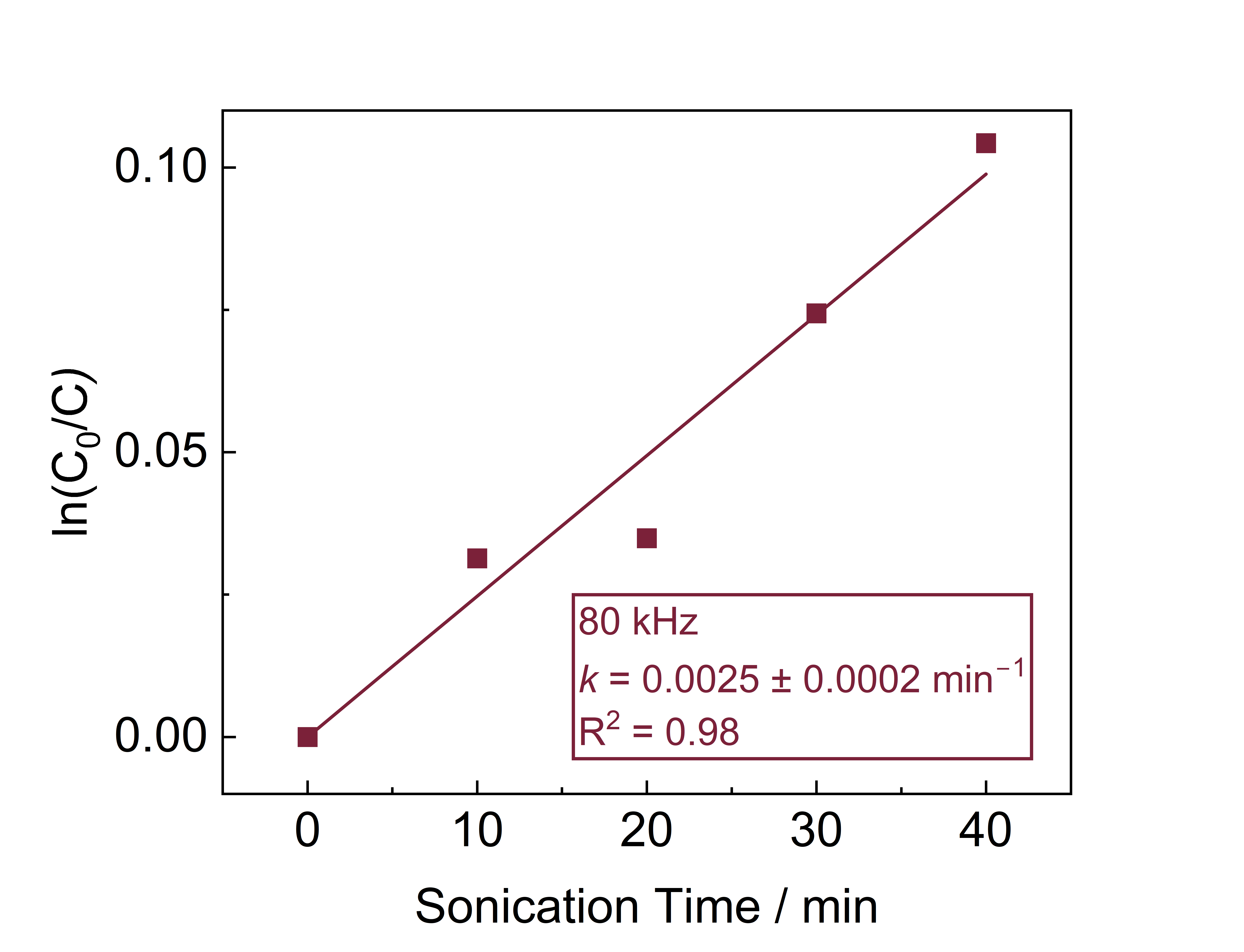

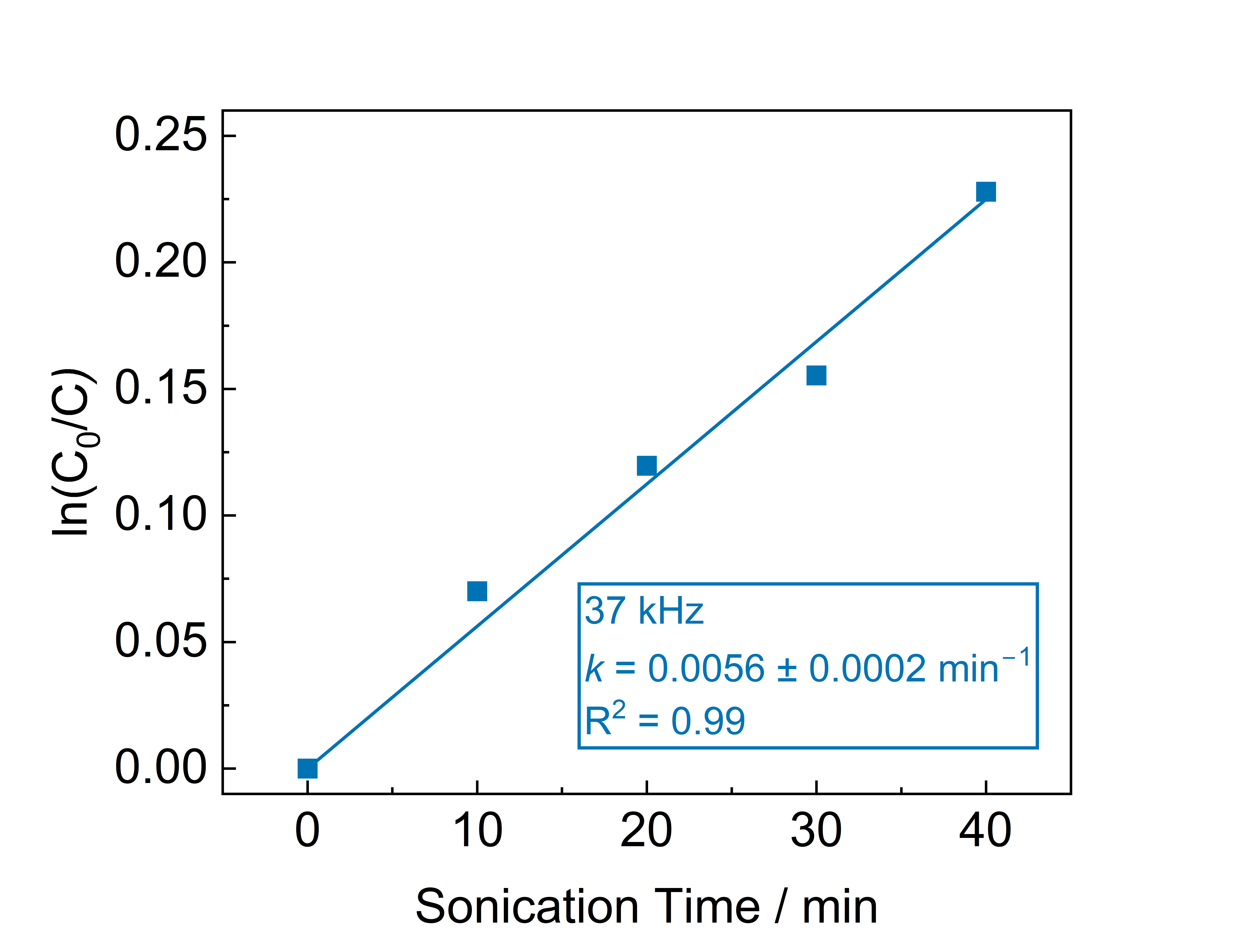
*

**Supplementary Figure 6:** *Single frequency degradation rate analysis.*

*Pseudo first-order rate analysis for the removal of bisphenol A by single frequency ultrasonic treatment. Rate constants have been calculated as the slope parameter from linear regression modelling of ln (C_0_/C) vs. time in each case, determined to be 0.0034 ± 0.0002 min^−1^ for 20 kHz ultrasound (a), 0.0056 ± 0.0002 min^−1^ for 37 kHz ultrasound (b), and 0.0025 ± 0.0002 min^−1^ for 80 kHz ultrasound.*

*
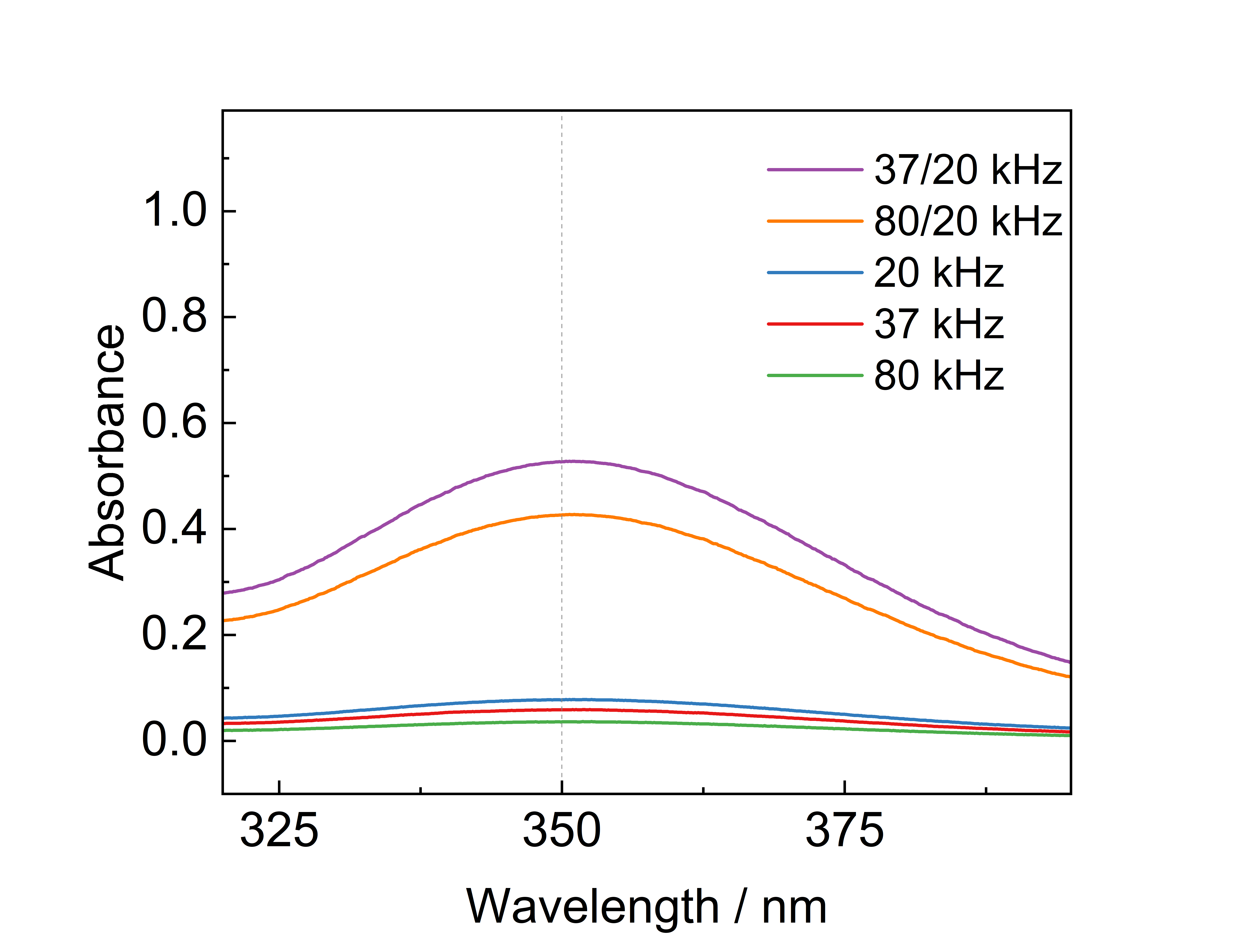
*

**Supplementary Figure 7:** *Iodide dosimetry of sonoreactor***.**

*The average UV-visible spectra for 0.1 M potassium iodide solutions following sonication for 5 mins with 80 kHz (green), 37 kHz (red), 20 kHz (blue), 80/20 kHz (orange), and 37/20 kHz (purple) frequencies are plotted vs. wavelength. The absorbance at λ = 350 nm was used to quantify the concentration of triiodide formed via oxidation of iodide by in situ generated reactive oxygen species.*

*
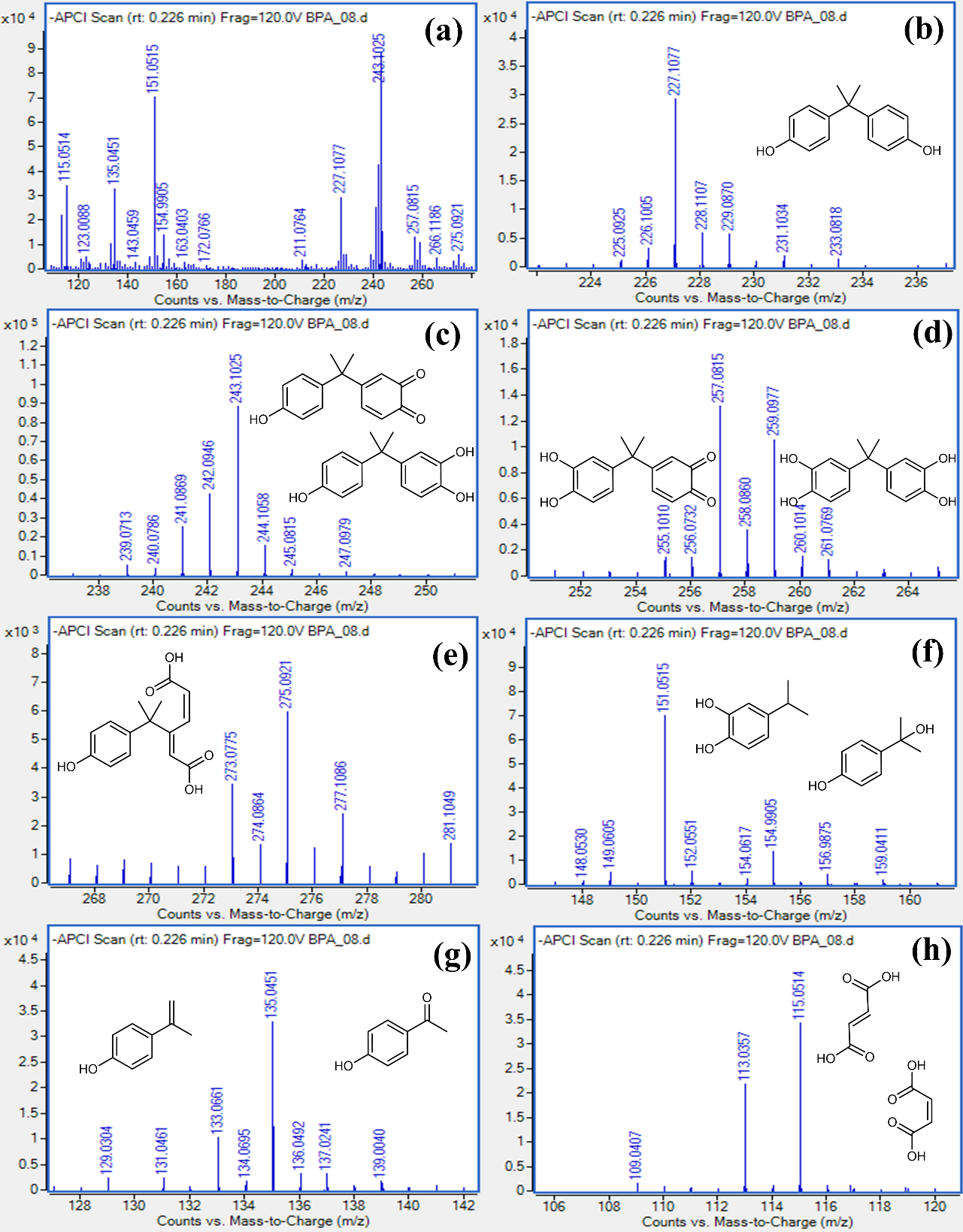
*

**Supplementary Figure 8:** *Mass spectrometry of degraded bisphenol A***.**

*Full mass spectrum of degraded BPA (after 37/20 kHz irradiation for 40 mins) (a), with selected portions of the spectrum enlarged to show the molecular ion (m/z = 227) (b), in addition to the proposed oxidative degradation products with m/z = 241 and 243 (c), m/z = 257 and 259 (d), m/z = 275 (e), m/z = 151 (f), m/z = 133 and 135 (g), and m/z = 115 (h). The most likely structures of the molecules corresponding to the observed signals are inset for reference.*
